# Multiple Acyl-CoA Dehydrogenase Deficiency with Variable Presentation Due to a Homozygous Mutation in a Bedouin Tribe

**DOI:** 10.3390/genes12081140

**Published:** 2021-07-28

**Authors:** Orna Staretz-Chacham, Shirly Amar, Shlomo Almashanu, Ben Pode-Shakked, Ann Saada, Ohad Wormser, Eli Hershkovitz

**Affiliations:** 1 Metabolic Clinic, Pediatric Division, Soroka University Medical Center, Beer Sheva 84101, Israel; 2 Faculty of Health Sciences, Ben-Gurion University, Beer Sheva 84101, Israel; elih@bgu.ac.il; 3 Genetic Lab, Soroka University Medical Center, Beer Sheva 84101, Israel; shirlyam@clalit.org.il; 4 National Newborn Screening Program, Ministry of Health, Tel-HaShomer, Ramat Gan 52621, Israel; shlomo.almashanu@sheba.health.gov.il; 5 Metabolic Disease Unit, Edmond and Lily Safra Children’s Hospital, Sheba Medical Center, Tel-Hashomer, Ramat Gan 52621, Israel; ben_pode@hotmail.com; 6 Sackler Faculty of Medicine, Tel-Aviv University, Tel-Aviv 39040, Israel; 7 Hadassah Medical Center, Department of Genetics, Jerusalem 911201, Israel; annsr@hadassah.org.il; 8 Faculty of Medicine, Hebrew University of Jerusalem, Jerusalem 911201, Israel; 9 The Morris Kahn Laboratory of Human Genetics, National Institute for Biotechnology in the Negev and Faculty of Health Sciences, Ben Gurion University of the Negev, Beer Sheva 84101, Israel; wormser@post.bgu.ac.il; 10 Department of Pediatrics D, Soroka Medical Center, Beer Sheva 84101, Israel

**Keywords:** multiple acyl-CoA dehydrogenase deficiency (MADD), electron-transfer flavoprotein (ETF), electron-transfer flavoprotein dehydrogenase (ETFDH), genotype, phenotype

## Abstract

Multiple acyl-CoA dehydrogenase deficiency (MADD) is a fatty acid and amino acid oxidation defect caused by a deficiency of the electron-transfer flavoprotein (ETF) or the electron-transfer flavoprotein dehydrogenase (ETFDH). There are three phenotypes of the disease, two neonatal forms and one late-onset. Previous studies have suggested that there is a phenotype–genotype correlation. We report on six patients from a single Bedouin tribe, five of whom were sequenced and found to be homozygous to the same variant in the *ETFDH* gene, with variable severity and age of presentation. The variant, NM_004453.3 (*ETFDH*): c.524G>A, p.(R175H), was previously recognized as pathogenic, although it has not been reported in the literature in a homozygous state before. R175H is located near the FAD binding site, likely affecting the affinity of FAD for EFT:QO. The single homozygous *ETFDH* pathogenic variant was found to be causing MADD in this cohort with an unexpectedly variable severity of presentation. The difference in severity could partly be explained by early diagnosis via newborn screening and early treatment with the FAD precursor riboflavin, highlighting the importance of early detection by newborn screening.

## 1. Introduction

Multiple acyl-CoA dehydrogenase deficiency (MADD) is a fatty acid and amino acid oxidation defect also known as glutaric aciduria type II. Its estimated prevalence is 1 in 750,000–2,000,000 newborns [1] with a much higher prevalence in China [2].

MADD affects mitochondrial fatty acid oxidation as well as the catabolism of branched amino acids, lysine and tryptophan [3]. The disease is caused by a deficiency of either electron-transfer flavoprotein (ETF) or electron-transfer flavoprotein dehydrogenase (ETFDH). ETF is composed of α and β subunits, encoded by the *ETFA* and *ETFB* genes, respectively, as well as electron-transfer flavoprotein-ubiquinone oxidoreductase (ETF-QO) [4,5], encoded by *ETFDH* [4,6]. MADD is inherited in an autosomal recessive manner.

There are three types of the disease based on clinical symptomatology: a neonatal-onset form with congenital anomalies (type I), a neonatal-onset form without congenital anomalies (type II) and a late-onset form (type III).

The two neonatal-onset forms are usually fatal, and affected patients develop severe non-ketotic hypoglycemia and metabolic acidosis with multisystem involvement accompanied by the excretion of large amounts of fatty acids and branched-chain amino acid metabolites [6]. Those with congenital anomalies usually succumb during the neonatal period, while those without anomalies die during the first months of life due to severe cardiomyopathy or, if they survive longer, tend to develop episodes of Reye syndrome-like decompensations [3].

The third or late-onset form has a wide spectrum of presentation, with either recurrent episodes, triggered by metabolic stress, of non-ketotic hypoglycemia, vomiting, lethargy, metabolic acidosis and hepatomegaly, or recurrent episodes of muscle involvement presenting as weakness and pain [3,6].

Olsen et al., among others, have previously reported a clear genotype–phenotype correlation with respect to disease severity [6,7].

We present here six patients from an extended Bedouin tribe, of which five were found to be homozygous to the same pathogenic variant c.524G>A, p.(R175H) in the *ETFDH* gene, with variable severity and age at presentation.

## 2. Materials and Methods

**Figure 1:**
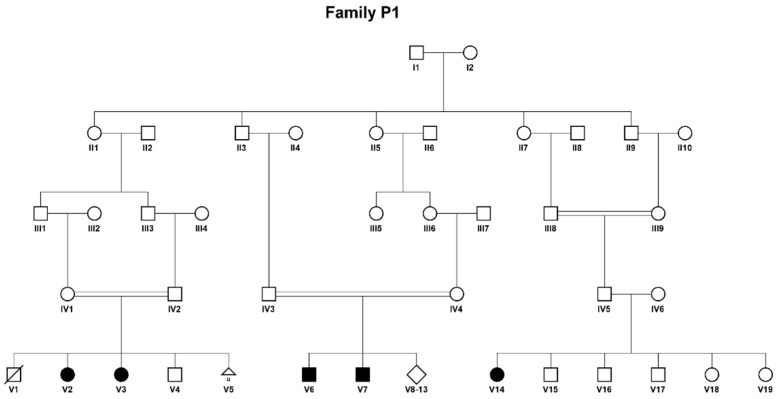
Pedigree of the consanguineous extended Bedouin family studied. Individuals affected with MADD are represented by full symbols. Patient V1 succumbed before molecular investigations were performed.

Clinical and biochemical details were collected from six patients, all from the same extended Bedouin family (Figure 1). Five of them were found to be homozygous to the same mutation in the *ETFDH* gene.

The automated extraction of DNA was performed using the QIAsymphony^®^ DSP DNA Midi Kit (Qiagen, Hilden, Germany) following the manufacturer’s recommendations. DNA was diluted in approximately 120 μL of buffer ATE. DNA concentration and quality was quantified using a Nanodrop ND-2000 spectrophotometer (Nanodrop Technologies, Wilmington, DE, USA).

Primer3 [8] was used for designing primers FW-AAAGTGTGACCATCAATGTAGCA REV-AAACAAAACTATACAAACCTCAGCAG for amplification of a 263 bp fragment of the *ETFDH* gene (NM_004453.3) containing the mutation c.524G>A; p.(R175H). PCR optimal annealing temperature was determined. Cycle sequencing was performed in both directions using amplification primers and BigDye Terminator v.1.1 kit (Life Technologies, Applied Biosystems, Carlsbad, CA, USA). Sequence purification was performed using BigDye XTerminator Purification kit (Life Technologies, Applied Biosystems, Foster City, CA, USA). The PCR plate was then sealed and vortexed for 30 min prior to being processed by an ABI 3500XL DNA analyzer (Life Technologies, Applied Biosystems, Foster City, CA, USA). Trace files analyses were performed using the Sequencing Analysis Software v.7 (Life Technologies, Applied Biosystems).

## 3. Results

### 3.1. Clinical Studies

**Figure 2:**
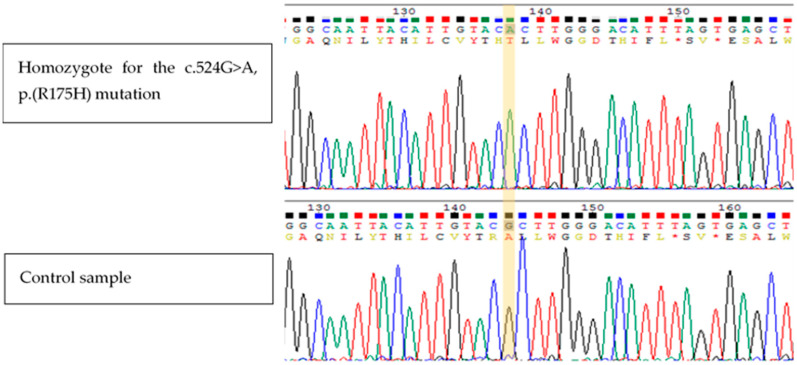
Sanger sequencing. Sanger sequencing for the NM_004453.3 (*ETFDH*): c.524G>A; p.(R175H) missense mutation of an affected individual and an unaffected unrelated individual.

We report six patients, all from the same extended Bedouin family (Figure 1). Three of the affected individuals are female (50%), and five of those available for molecular analysis were found to be homozygous to the same mutation in the *ETFDH* gene (Figure 2).

- Patient V1 (Figure 1) was the first son of consanguineous parents of Bedouin origin. He was born at term following an uneventful pregnancy with a birth weight of 3715 g and head circumference of 35 cm. The patient was discharged home after 2 days and died at home after two hours. Unfortunately, no investigations were performed.
- Patient V2 is a female, the second child in the same family. The pregnancy was uneventful, and she was born at term with a birth weight of 3570 g and head circumference of 34 cm. Apart from inspiratory stridor that resolved spontaneously, physical examination was considered normal. Due to the family history a metabolic work-up was performed immediately after birth (which was before the expanded newborn screening era in Israel) which resulted in the following findings: palmitate oxidation in lymphocytes was reduced to 40% in comparison to control, and acylcarnitine profile revealed increased levels of C4-C16 including C14 and C14:1, with the highest increase in C6-C10, accompanied by increased levels of glutarylcarnitine and isovalerylcarnitine (Table 1). These led to the suspected diagnosis of MADD. A urinary organic acids test at the time supported this clinical diagnosis. The patient was started on L-carnitine and riboflavin with normal development and no episodes of decompensation. The patient is now 16.5 years old and has been treated from birth with L-carnitine 50–100 mg/kg and riboflavin 100 mg.

- Patient V3 is a female, the third child in the same family. She was born at term after an uneventful pregnancy via cesarean section with a birth weight of 3610 g and head circumference of 36.5 cm. Newborn screening was positive twice and raised the suspicion of a MADD diagnosis, supported by a confirmatory metabolic work-up, including an acylcarnitine profile with increased C4-C10, C6 carnitines and upper range C5, C12-C18 and dicarboxylic aciduria with increased hexanoylglycine, glutaric, ethylmalonic and 2-OH-glutaric in urinary organic acids (Table 1). Due to suspected MADD, she was started on L-carnitine and riboflavin with normal development and no episodes of decompensation. The patient is now 10.5 years old and has been treated from birth with L-carnitine 50–100 mg/kg and riboflavin 100 mg.
- Patient V6 is a male, the first son in his nuclear family. He was born after an uneventful pregnancy to consanguineous parents of Bedouin origin with a birth weight of 4000 g and head circumference of 35 cm. At 9 months he was admitted due to encephalopathy after 4 days of diarrhea and refusal to feed and on physical examination had hepatomegaly, a micropenis and undescended testes. His viral panel, hepatitis A, B, and C serology, carcinoembryonic antigen (CEA) and α-fetoprotein were normal. Urine was negative for reducing substances. He was treated by intravenous D10 and electrolytes. On the fifth day, the patient deteriorated and developed hypoglycemia and hyperammonemia with elevated liver enzymes and normal serum bilirubin, prolonged coagulation tests and hyperlipidemia. A brain CT revealed mild frontal atrophy and ventriculomegaly. A liver biopsy demonstrated a Reye-like disease. Urinary organic acids showed increased glutaric- and β-OH-butyric acids. Total and free carnitine levels were low (Table 1). The patient improved with fresh frozen plasma, cryoprecipitate and total parenteral nutrition, and was started on L-carnitine with a diagnosis of fatty acid oxidation defect. At 16 years he developed recurrent perianal abscesses necessitating repeated drainage. The patient was treated from the age of 9 months until he was 18 years old with L-carnitine 50–100 mg/kg, though not consistently. He is currently 31 years old and is untreated due to lack of compliance.
- Patient V7 is a male, a sibling of patient V6 and the eighth child in the family. He was born after an uneventful pregnancy via cesarean section with a birth weight of 4280 g and head circumference of 37 cm. At 4 months he was admitted due to encephalopathy after 5 days of diarrhea and refusal to feed and on physical examination had hepatosplenomegaly accompanied by hypotonia. Laboratory work-up revealed hypoglycemia, elevated liver enzymes, elevated serum lactate, hypertriglyceridemia and thrombocytopenia. A viral panel and hepatitis A, B, and C serology were normal. Acylcarnitine profile showed a mild increase of C4–C10 and a marked increase of C12–C18, and urinary organic acids demonstrated massive excretion of dicarboxylic acids, increased lactic-, glutaric-, ethylmalonic-, p-OH-phenyl-lactic acids (Table 1). Total and free carnitine levels were low. He was treated by D10 and electrolyte infusion and was started on carnitine under a diagnosis of fatty acid oxidation defect. The patient has had recurrent admissions due to metabolic decompensations. He is currently 17 years old and has been treated from the age of 4 months with L-carnitine 100 mg/kg and later, due to low compliance, with 50 mg/kg.
- Patient V14 is a female, born at term after an uneventful pregnancy to consanguineous parents of Bedouin origin. She presented for the first time at the age of 19 years with muscle weakness, fatigue and abdominal pain for two months accompanied by weight loss. She was admitted due to inability to walk, rhabdomyolysis and elevated liver enzymes which resolved after D10 and electrolyte infusion, and due to suspected MADD was also started on high dose of L-carnitine and riboflavin. Metabolic work-up revealed increased C4–C16:1 in acylcarnitine profile and dicarboxylic aciduria with increased secretion of hexanoylglycine, glutaric-, ethylmalonic-, glutaric-, adipic-, isovaleric-, suberyl, 2-methyl butyry-acids on urinary organic acids profile (Table 1), which confirmed the diagnosis of MADD. The patient is 20 years old and has been treated with L-carnitine 50 mg/kg and riboflavin 100 mg for the last 14 months since her diagnosis and is currently stable with no admissions under this treatment.

### 3.2. Genetic Studies

We found a missense mutation in NM_004453.3 (*ETFDH*): c.524G>A; p.(R175H), designated in ClinVar as VCV000031576, and in dbSNP as rs121964955 (multiallelic). This variant was classified as likely pathogenic by Franklin by Genoox (https://franklin.genoox.com, accessed on February 2021) with population frequency of less than 0.01% according to gnomAD (last accessed July 2021, [9]), and has been previously interpreted as pathogenic, as it was reported in a compound heterozygous form with other pathogenic variants in several manuscripts [10,11,12,13,14,15] in affected patients. Moreover, two other reported variants affect the same amino acid, c.524G>C; p.(R175P) and c.524G>T; p.(R175L) (VCV000203713 and VCV000012029 accordingly), and are also interpreted as pathogenic.

The pathogenicity of the variant is supported by multiple prediction algorithms: PolyPhen2 prediction: “Probably damaging” (1), SIFT prediction: “Damaging” (0), MutationTaster prediction: “Disease causing” (1), and CADD score: 35 [16,17,18,19]. This missense mutation replaces arginine with histidine at codon 175 of the ETFDH protein. The arginine residue is highly conserved among vertebrates; UCSC’s Multiz alignments of 100 vertebrate species indicate all have arginine at this position (as presented on UCSC’s genome browser) [20,21]. Thus, the physicochemical difference between arginine and histidine is likely to exert a detrimental effect on the protein.

## 4. Discussion

MADD is a rare, potentially treatable β-oxidation disorder with three main subtypes: types I/II represent early onset in the neonatal period or infancy and type III represents the late onset of the disease [3,22].

The most common etiology of MADD is mutations in the *ETFDH* gene, which encodes the electron transfer flavoprotein dehydrogenase (ETFDH) [23,24], relative to which most cases are associated with late onset or RR-MADD (riboflavin responsive-MADD) as is the case for the patients reported in this study.

Fan et al. have shown that the ETF-QO protein is integrated in the inner mitochondrial membrane and its crystal structure is comprised of three domains: the FAD domain, the 4Fe4S cluster domain and the UQ-binding domain [11,25]. The R175 amino acid is located near the FAD binding site; thus, the mutant p.(R175H) would likely affect the binding affinity of FAD to ETF:QO, and may reduce the activity of ETFDH, as also predicted by PolyPhen2 [11,17]. Interestingly, the p.(R175H) variant was present in the heterozygous state in the gnomAD database, with a frequency of 4/246132 chromosomes, and in a compound heterozygous state in other reported patients [10,11,12,13,14,15]. Here we report this variant for the first time in a homozygous state. Although it might be expected to present with a homogenous, severe phenotype our patients presented with a variety of ages of onset and severity.

During acute decompensations, urinary organic acids are expected to show dicarboxylic aciduria and the accumulation of marker metabolites of the blocked enzymes, while between these episodes we expect biochemical tests to be much improved or even normalized. The plasma acylcarnitine profile is also expected to show a characteristic picture of an increase in all chain acylcarnitines [3]. Therefore, while patients V2 and V3 did not present clinically in the neonatal period, their laboratory and newborn screening (NBS) results support an early decompensation. Further deterioration was avoided due to the establishment of early treatment, and decompensations were absent thereafter due to compliance with treatment, in addition to significant improvement in the acylcarnitine profile under treatment. This is in accord with Grünert et al., who emphasized that diagnosis may be delayed because most patients do not display typical biochemical patterns of urine organic acids and blood acylcarnitines during times of wellbeing [26].

Presently, three of the living patients are riboflavin responders and with proper treatment do not have recurrent episodes.

Closer prenatal follow-up and appropriate genetic consultation after the delivery or diagnosis of either patient V2 or V6 is warranted, especially with the consanguinity in this extended Bedouin family. However, due to cultural beliefs no genetic consultation was performed before patient V3 was born, and she was diagnosed by NBS with a high index of suspicion, like patient V14, who was born before the extended Israeli NBS was started and presented later in life [27].

Schiff et al. have reported that patients surviving the early decompensations of MADD type II will develop Reye syndrome-like decompensations later in life, as did our patients V6 and V7, who therefore partially fit the type II pattern, although their survival does not, due to the reported short life span. MADD type III patients are expected to present with a variety of symptoms such as intermittent episodes of vomiting, hypoglycemia and metabolic acidosis during infancy or episodic muscular weakness during adulthood, up to rhabdomyolysis [3] as reported for patient V14. Additionally, all our patients were homozygous to the R175H mutation, which is located in the FAD-binding domain and expected to result in total loss of protein [28], emphasizing the importance of early treatment for changing the natural history. While this is the case for patients V2 and V3, patients V6 and V7 have had longer than expected life spans and are on no or low treatment, which could partially be explained by nutrition or other external factors.

Grünert et al. reviewed the literature and concluded that most patients with adult onset harbor mutations in the *ETFDH* gene and that majority of them are riboflavin-responders [26]. However, since in times of well-being biochemical patterns of organic acids and acylcarnitine in urine are expected to be normal, we assume that when NBS is slightly abnormal MADD should be suspected, even if patients are clinically asymptomatic, as in our patients, and molecular testing should be pursued accordingly. As described in the literature there are a number of inborn errors of metabolism, such as medium-chain acyl-CoA dehydrogenase deficiency and very-long-chain acyl-CoA dehydrogenase deficiency, that by biochemical diagnosis through NBS may have an improved prognosis with good compliance, leading to the prevention of metabolic crises and the avoidance of clinical symptomatology in some patients [29,30].

Reports on cellular studies performed by Cornelius et al. have shown that depending on the localization of the mutation, the increased concentration of FAD caused by the addition of riboflavin results either in improved cellular activity or does not make a change, depending on whether the mutation involved the FAD-binding domain with an altered conformation of the interaction site [31]. Although the reported mutation in our manuscript is indeed located on the FAD-binding domain, under riboflavin treatment patient V14 experienced complete recovery. On biochemical analysis there were low levels of free carnitine during metabolic decompensations as reported in the literature, and also in patients V6 and V7, making it an important treatment for these patients. By functional and biochemical studies, it was further suggested that CoQ10 could also contribute to increased ETF-QO stability and add to the restoration of protein function, and it is therefore suggested as a supplementary treatment for MADD [28]. Since MADD results from the impaired oxidation of fatty acids and catabolism of branched-chain amino acids, we suggest the benefit of a low-fat and -protein diet [28].

Riboflavin treatment is thought to promote FAD-binding to ETF-QO mutants by stabilizing the mutant conformations [7,31,32,33]. Patients V2 and V3 are siblings of patient V1, who died at the age of 2 days before diagnosis. They were both diagnosed at birth with no reported decompensations under treatment with carnitine (50–100 mg/Kg/day) and riboflavin (100 mg/day), emphasizing the importance of early treatment and its effectiveness, especially when compared to patients V6 and V7, who have experienced recurrent decompensations and a lack of compliance.

Different studies have reported a genotype–phenotype correlation in MADD, especially in severe cases, associated with null mutations severely affecting mRNA expression, processing and/or stability. These were reported with regard to type III, where the mutated gene is expected to be *ETFDH*, types I/II with *ETFA* or *ETFB* variants or with regard to specific pathogenic mutations [6,15,26]. However, our experience with the patients reported herein, as summarized in Table 1, contradicts a genotype–phenotype correlation, since all our patients are homozygous to the same mutation, but vary in presentation from neonatal biochemical presentation to neonatal onset (3/6; 50%) (including the sibling who died two days after birth and is suspected to have been affected), to early infancy (2/6; 33%) and one that presented as a clear late onset. Therefore, the reported patients do not support a genotype–phenotype correlation in MADD. However, the absence of any decompensations throughout life with proper treatment in patients V2 and V3 emphasizes the importance of NBS and adherence to management with L-carnitine and riboflavin.

## 5. Conclusions

As reported in our patients early biochemical diagnosis by NBS with good compliance may lead to prevention of metabolic crises. Therefore, we suggest that not only a combination of clinical symptomatology and biochemical results but also biochemical results by themselves should be part of appropriate disease grading.

We conclude that there are no absolute genotype–phenotype correlations in MADD, and that early treatment and compliance in patients with *ETFDH* mutations results in better prognosis and fewer decompensations.

## Figures and Tables

**Table 1:** Patients’ biochemistry results.

Patient	Urinary Organic Acids	Acyl Carnitines	Free Carnitine (Norm: 25–45 mmol/L)	Total Carnitine (Norm: 30–50 mmol/L)
V2	Increased hexanoylglycine, glutaric, ethylmalonic, 2-OH- glutaric	Increased C3/C5, C8, C10, C10:1, C12, C14, C14:1/C16 and glutarlcarnitine, isovalerylcarnitine	NA	NA
V3	Dicarboxylic aciduria, mild ketonuria, increased hexanoylglycine, glutaric, ethylmalonic, 2-OH- glutaric	Increased C4, C6, C8, C10, C6 dicarboxylic; upper range C5, C12, C14, C16, C18	37.3	60.5
V6	NA	Mild increase C4–C18	Low: 12 nmol/mL	NA
V7	NA	Mild increase of C4–C10 and marked increase C12–C18	Low: <10 nmol/mL	NA
V14	Dicarboxylic aciduria, increased hexanoylglycine, glutaric, ethylmalonic, glutaric, adipic, isovaleryl, suberyl, 2-methyl butyryl	Increased C4, C6, C8, C10, C12, C14, C14:1, C14:2, C16, C16:1	NA	NA

NA: not available.

## Data Availability

The data that support the findings of this study are available on request from the corresponding author. The data are not publicly available due to privacy or ethical restrictions.
